# Millimeter-Size Spherical Polyurea Aerogel Beads with Narrow Size Distribution

**DOI:** 10.3390/gels4030066

**Published:** 2018-08-06

**Authors:** Despoina Chriti, Grigorios Raptopoulos, Maria Papastergiou, Patrina Paraskevopoulou

**Affiliations:** Laboratory of Inorganic Chemistry, Department of Chemistry, National and Kapodistrian University of Athens, Panepistimiopolis Zografou, 15771 Athens, Greece; chritides@chem.uoa.gr (D.C.); grigorisrap@chem.uoa.gr (G.R.); mapapast@chem.uoa.gr (M.P.)

**Keywords:** aerogels, Desmodur N3300, ethylenediamine, polyurea, spherical beads

## Abstract

We report the room temperature synthesis of spherical millimeter-size polyurea (PUA) aerogel beads. Wet-gels of said beads were obtained by dripping a propylene carbonate solution of an aliphatic triisocyanate based on isocyanurate nodes into a mixture of ethylenediamine and heavy mineral oil. Drying the resulting wet spherical gels with supercritical fluid (SCF) CO_2_ afforded spherical aerogel beads with a mean diameter of 2.7 mm, and a narrow size distribution (full width at half maximum: 0.4 mm). Spherical PUA aerogel beads had low density (0.166 ± 0.001 g cm^–3^), high porosity (87% *v*/*v*) and high surface area (197 m^2^ g^–1^). IR, ^1^H magic angle spinning (MAS) and ^13^C cross-polarization magic angle spinning (CPMAS) NMR showed the characteristic peaks of urea and the isocyanurate ring. Scanning electron microscopy (SEM) showed the presence of a thin, yet porous skin on the surface of the beads with a different (denser) morphology than their interior. The synthetic method shown here is simple, cost-efficient and suitable for large-scale production of PUA aerogel beads.

## 1. Introduction

Aerogels are highly porous ultralight materials, consisting of low-density 3D assemblies of nanoparticles [1,2]. Formally, they can be defined as solid colloidal or polymeric networks of particles expanded throughout their entire volume by a gas [3,4]. They are formed by removing all swelling agents from a wet-gel without substantial volume reduction or network compaction. The latter is accomplished by drying wet-gels by turning the solvent filling the pores into a supercritical fluid that is released like a gas. In essence, by that method, aerogels retain the structural shape of their wet-gel precursors. [2]. Several types of aerogels have been reported, including inorganic [5,6,7,8,9], organic (based on biopolymers [10,11,12,13] or synthetic polymers [14,15,16,17]), and hybrid inorganic/organic [18,19,20,21,22].

Among the synthetic polymer aerogels, polyurea (PUA) aerogels were first reported in a 1996 U.S. Patent [23] using the traditional method of urea synthesis, i.e., nucleophilic addition of amines to isocyanates (Equation (1)). A few years later, an alternative method was proposed, both more cost-efficient and more environmentally friendly, which replaced the amines with water [17]. According to that method, water reacts with isocyanates in the presence of triethylamine as catalyst, forming carbamic acids, which decompose to form carbon dioxide and amine, which in turn reacts rapidly with yet unreacted isocyanate to form urea (Equation (2)).

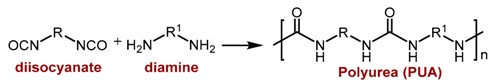
(1)

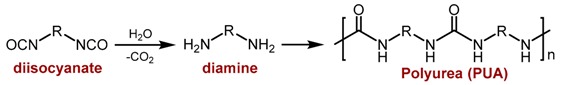
(2)

To the best of our knowledge, only two examples of PUA aerogels in the form of particles have ever been reported. The beads of this study are very different from both.

PUA aerogel powders have been prepared from the reaction between tetrakis(4-aminophenyl)methane and various alkyl diisocyanates (1,4-diisocyanatobutane, hexamethylene diisocyanate, toluene 2,4-diisocyanate, 1,8-diisocyanatooctane, 1,12-diisocyanatododecane) in dimethylformamide (DMF) at room temperature, followed by precipitation by the addition of acetone as a non-solvent [24]. The product consisted of spherical particles, the size of which (on the order of hundreds of nm) was larger than the gel network nanoparticles (about 9–30 nm, measured with dynamic light scattering), and depended on the chemical identity of the diisocyanate; the longer the aliphatic chain of the isocyanate, the larger the particles obtained. Brunauer–Emmett–Teller (BET) surface areas measured with N_2_ sorption were in the range of 19–68 m^2^ g^–1^.

PUA aerogel powders have also been produced by mechanical agitation (at 30 °C) of sols from toluene 2,4-diisocyanate (2,4-TDI) and 4,4’-oxydianiline (4,4’-ODA) in acetone or acetonitrile [25]. The reaction was always run under conditions of precipitation polymerization. The typical total monomer concentration (2,4-TDI + 4,4’-ODA) in the sol was 1% *w*/*w*. Resulting powders were compared among themselves, i.e., with no reference to monolithic aerogels. Precipitates were dried at 60 °C to powdery products. Nanomorphology, however, was a complicated function of several parameters: for example, with no stirring at all, the network nanomorphology changed from fibrous to particulate (granular aggregates) by lowering the reaction temperature from 30 to 0 °C. Similarly, using reciprocated stirring, nanomorphology changed from fibrous to particulate by increasing the total monomer concentration; nanofibers were obtained with monomer concentrations lower than 2% *w*/*w*. Running the reaction in acetonitrile yielded large (5–20 μm in diameter) spherical particles rather than fibers. Switching from fibers in acetone [17] to large micron-size particles in acetonitrile [26] has been also reported with monolithic PUA aerogels synthesized from the same monomer employed in this study, that is Desmodur N3300 and water.

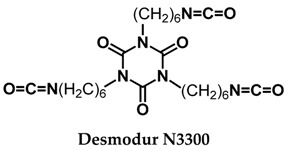



In this work, we report the first to our knowledge synthesis of spherical millimeter-size PUA aerogel beads. These beads were prepared via the dripping method, by the reaction of an aliphatic triisocyanate (Desmodur N3300) and ethylenediamine according to Equation (1). The two reagents were introduced in two immiscible phases, propylene carbonate and mineral oil, respectively. The PUA beads were characterized in terms of chemical structure by IR and ^13^C cross-polarization magic angle spinning (CPMAS) NMR spectroscopy, and in terms of material properties by measurements of density, porosity and BET surface area, while their morphology was studied with scanning electron microscopy (SEM) and their thermal stability with thermogravimetric analysis (TGA). Indistinguishable beads in terms of chemical composition, physical and morphological properties were obtained from control experiments in which a stoichiometric amount of water was added deliberately in the Desmodur N3300/propylene carbonate phase. The synthetic method presented herewith is one-step, uses inexpensive reagents, and is suitable for large-scale production of PUA aerogel beads.

## 2. Results and Discussion

### 2.1. Synthesis of Spherical PUA Aerogel Beads

**Figure 1:**
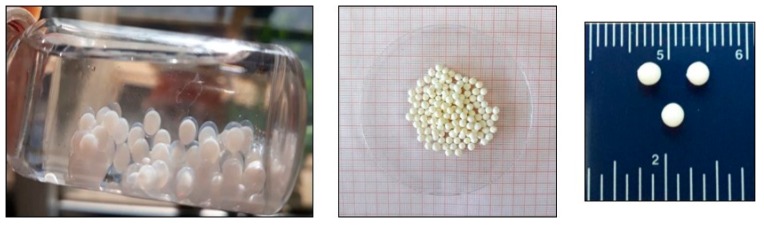
Spherical polyurea (PUA) aerogel beads during solvent exchange with acetone (**left**) and after dried with supercritical fluid (SCF) CO_2_ (**middle** and **right**).

A propylene carbonate solution of Desmodur N3300 was added dropwise using a disposable pipette into a graduated cylinder containing a mixture of ethylenediamine and mineral oil. Spherical wet-gel beads were formed immediately upon addition, and settled at the bottom of the cylinder. They were left in that condition for aging for 15 min. Subsequently, they were removed from the mineral oil, solvent-exchanged with acetone (Figure 1 **left**) and were dried in an autoclave with supercritical CO_2_ into spherical PUA aerogels (Figure 1 **middle** and **right**). Alternatively, mineral oil was replaced with water, but the beads did not remain spherical and the resulting aerogels had very low surface areas.

### 2.2. General Material Properties of Spherical PUA Aerogel Beads

**Figure 2:**
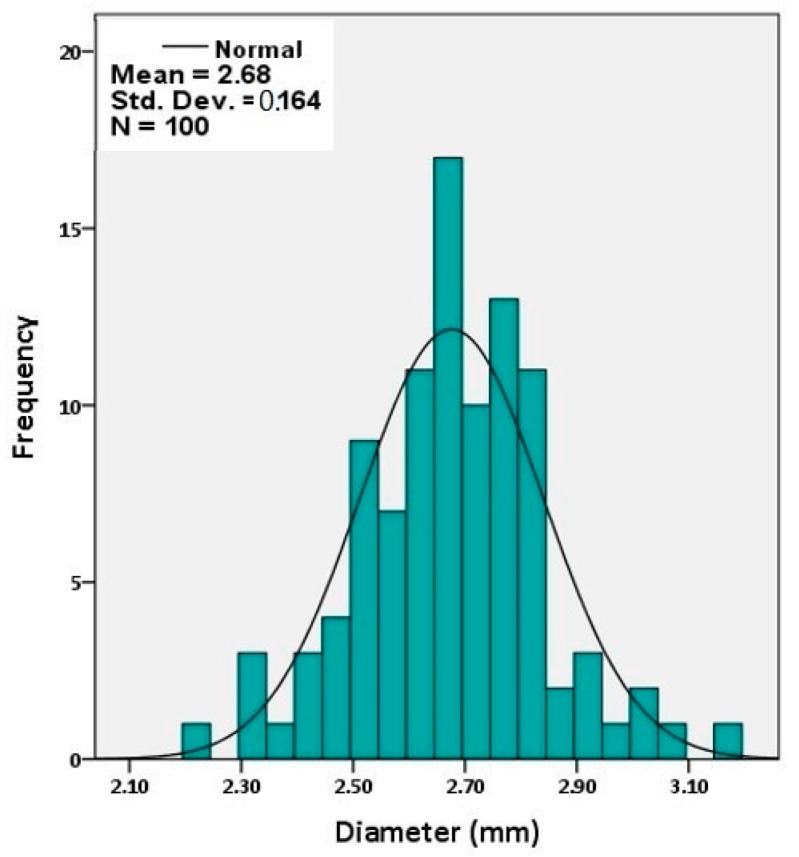
Size distribution of spherical PUA aerogel beads (calculated using IBM SPSS Statistics 24). Sample size: 100 beads.

**Figure 3:**
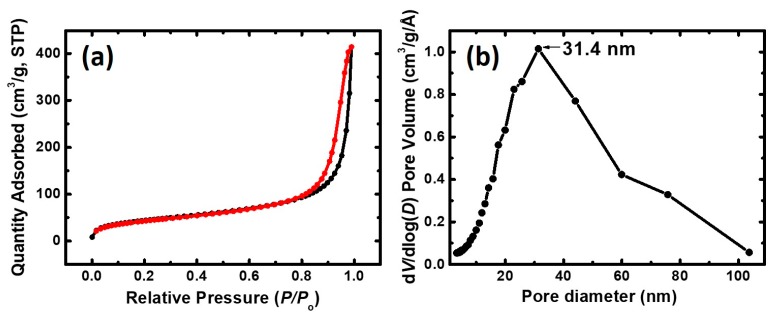
(**a**) N_2_-sorption isotherm for spherical PUA aerogel beads; (**b**) Barrett–Joyner–Halenda (BJH) pore size distribution.

The spherical aerogel beads had a mean diameter of 2.7 mm, a narrow size distribution (full width at half maximum: 0.4 mm, Figure 2), low density (0.166 ± 0.001 g cm^–3^), and they were porous (87% *v*/*v*) with high surface area (197 m^2^ g^–1^). These properties were similar to the properties of PUA aerogel monoliths prepared with the same concentration of Desmodur N3300 and a stoichiometric amount of water in acetone according to Equation (2) (Table 1) [17]. It is noted that the larger particles and lower surface areas observed for monoliths in propylene carbonate is attributed to the lower amount of catalyst in the propylene carbonate sols relative to acetone. At any rate, it is pointed out that the chemical compositions of PUA aerogels formed with ethylenediamine (PUA-A) or water (PUA-B) were different, as discussed in Section 2.3 below. The porous network of the aerogel beads was probed with N_2_-sorption porosimetry. As shown in Figure 3, the N_2_-sorption isotherm increased rapidly above *P*/*P*_o_ = 0.9 and exhibited narrow hysteresis loops, indicating a macroporous material with some mesoporosity. Indeed, by comparing pore volumes (Table 1), *V*_Total_ > *V*_1.7–300 nm_ (where *V*_Total_ was calculated from bulk and skeletal densities, and *V*_1.7–300 nm_ was obtained from N_2_ sorption). For pores falling in the range of 1.7–300 nm, the Barrett–Joyner–Halenda (BJH) curves showed a maximum at 31.4 nm (inset in Figure 3). That pore size distribution was rather broad as expected for networks formed via particle aggregation processes.

**Figure 4:**
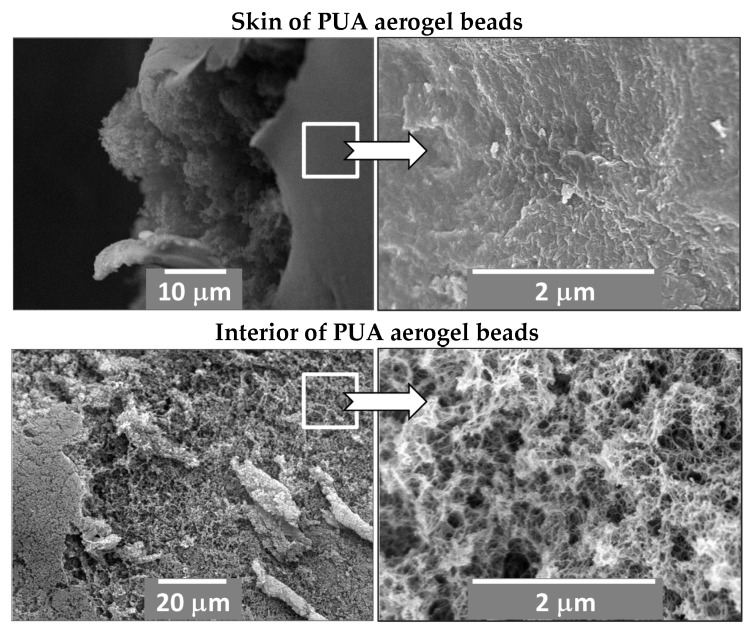
Scanning electron microscopy (SEM) images of spherical PUA aerogel beads at different magnifications: skin (**top** row) and interior (**bottom** row).

SEM images (Figure 4) showed the presence of a thin skin on the surface of the beads with a different morphology than their interior. The interior of the beads consisted of entangled fibers, while the skin appeared to be consisting of dense agglomerations of fused particles. Yet, the skin was porous because it allowed gasses to penetrate the interior, i.e., during N_2_-sorption porosimetry. According to the literature [17], a similar switch from fibrous to dense particular morphology is observed in monolithic PUA aerogels obtained via Equation (2), when the Desmodur N3300/H_2_O concentration in the sol and consequently the gelation rate increases. Consistent with those observations, at the interface of the propylene carbonate droplet and the surrounding mineral oil, the ethylenediamine concentration is high, and therefore its reaction with Desmodur N3300 is fast and creates the particulate crust of the beads. In the interior of the beads, diffusion of ethylenediamine establishes a concentration gradient that slows the reaction down, leading to the fibrous morphology that is observed. It is worth noting that the bulk density of the PUA beads in this study (0.166 g cm^–3^) was in the range (0.13–0.19 g cm^–3^) where monoliths undergo their morphological switch from fibrous to particulate as the reaction (gelation) rate increases [17,27].

### 2.3. Chemical Characterization of Spherical PUA Aerogel Beads

**Scheme 1:**
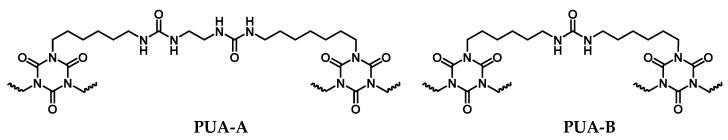
Structure of PUA obtained from the reaction of Desmodur N3300 and ethylenediamine in propylene carbonate (PUA-A) and from the reaction of Desmodur N3300 and water (PUA-B).

Despite similar physical properties, the chemical composition of PUA monoliths obtained from Desmodur N3300/H_2_O using triethylamine as catalyst via Equation (2) (Scheme 1, PUA-B) was different from the structure of PUA beads obtained from Desmodur N3300/ethylenediamine (Scheme 1, PUA-A), which underlines the fact that material properties (such as the bulk density, the BET surface area and porosity) are controlled by nanostructure (e.g., fibers vs. particles) rather than by minor differences at the molecular level. For example, the nanostructure of the beads is similar to the nanostructure of monoliths obtained from acetone, and the material properties of the two materials follow one another closely. The reaction of the triisocyanate with water, in the presence of triethylamine as catalyst, was carried out according to the procedure in the literature [17], using propylene carbonate instead of acetone. The reaction was much faster in propylene carbonate than in acetone, and therefore the amount of catalyst was reduced in order to slow it down and obtain well-shaped monoliths. Similarly, the reaction of Desmodur N3300 with ethylenediamine was extremely fast and vigorous to the point that no well-shaped monoliths could be obtained. Furthermore, when a stoichiometric amount of water was added in the propylene carbonate solution of Desmodur N3300, the resulting beads were identical to the ones obtained without water. Of course, that was not surprising because amines are better nucleophiles than water [28], and in addition, ethylenediamine was in large excess compared to water and reacts vigorously with the triisocyanate.

**Figure 5:**
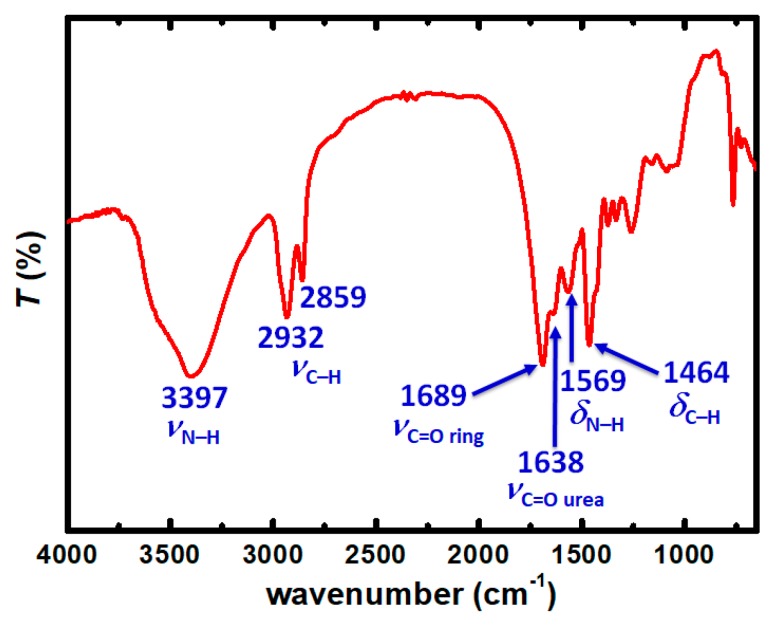
FT-IR spectrum of spherical PUA aerogel beads.

**Figure 6:**
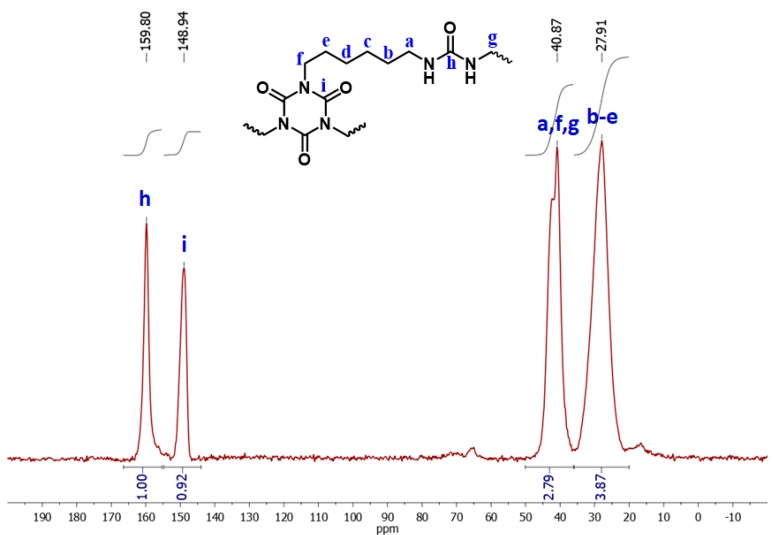
^13^C cross-polarization magic angle spinning (CPMAS) NMR spectrum of spherical PUA aerogel beads.

**Figure 7:**
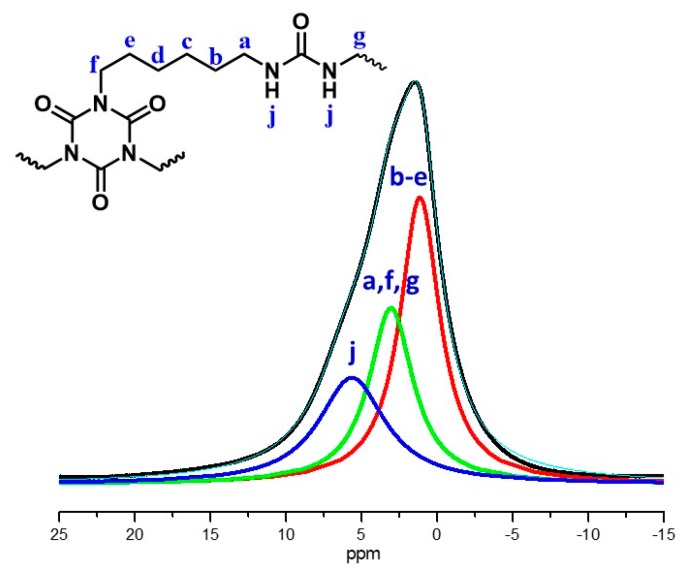
^1^H magic angle spinning (MAS) NMR spectrum of spherical PUA aerogel beads. Experimental spectrum (black line), fitted peaks (blue, green and red lines) and total fit (cyan line).

Chemical characterization of the beads was carried out with FT-IR (Figure 5), ^13^C CPMAS NMR (Figure 6) and ^1^H magic angle spinning (MAS) NMR (Figure 7). The stretching vibration of the carbonyl groups of the isocyanurate ring appears at 1689 cm^–1^ (Figure 5). The stretching vibration of N–H appeared at 3397 cm^–1^ and the bending vibration of N–H at 1569 cm^–1^. Those peaks are in agreement with the expected polymer structure and with the literature [17]. In the solid-state ^13^C CPMAS NMR spectrum (Figure 6), there are two peaks corresponding to the two different carbonyl groups; the peak at 159.8 ppm corresponds to the carbonyl of the urea, and the peak at 148.9 ppm corresponds to the carbonyl of the isocyanurate ring. The peaks in the aliphatic region, at 40.9 and 27.9 ppm, can be attributed to the –CH_2_– groups of the polymers, with the –CH_2_– groups adjacent to –NH groups appearing downfield. The absence of a peak at 120 ppm, attributed to the isocyanate group of Desmodur N3300 [17], indicated that there are no unreacted NCO groups in the polymer. Integration of the two carbonyl peaks gives a ratio of 1:0.92 (theoretical: 1:1), and integration of the two aliphatic peaks gives a ratio of 1:1.39 (theoretical: 1:1.33), both in agreement with the structure of the polymer (PUA-A). In comparison to the literature spectrum for PUA synthesized from Desmodur N3300 and water (PUA-B) [17], the chemical shifts are practically identical, but the integral ratios are different. The integration of the carbonyl peaks gives a ratio of 1:1.87 (theoretical: 1:2), and integration of the two aliphatic peaks gives a ratio of 1:2.35 (theoretical: 1:2), as was expected from the structure of PUA-B (Figure S1). The solid-state ^1^H MAS NMR spectrum (Figure 7) also supports the expected structure. The experimental spectrum consists of one broad peak at 1.48 ppm (black line), which was fitted to three peaks, centered at 5.46 (blue line), 2.97 (green line) and 1.13 ppm (red line). Those peaks can be attributed to –NH, –N–CH_2_– and –C–CH_2_– protons, respectively.

**Figure 8:**
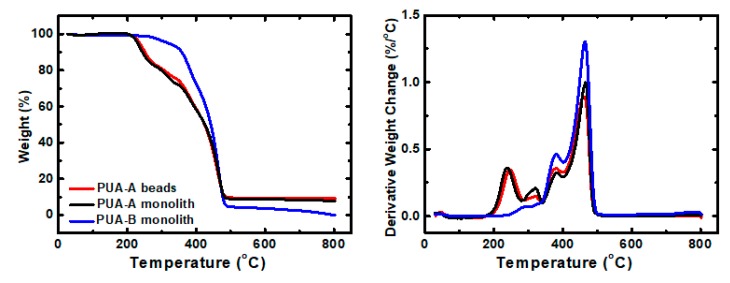
Weight loss as a function of temperature (**left**) and derivative weight loss with temperature (**right**) of spherical PUA aerogel beads and PUA monoliths synthesized in propylene carbonate using the same monomer concentration, as indicated.

Thermogravimetric analysis of spherical PUA aerogel beads, as well as the corresponding monolith (PUA-A) and the monolith synthesized from the reaction of Desmodur N3300 with water (PUA-B), was carried out under N_2_ (Figure 8; Table 2). In all cases, decomposition took place in more than one step, indicating a complex mechanism, and the final residue was negligible. Both PUA-A and PUA-B materials showed two major decomposition events at 381–382 °C and 463–466 °C. PUA-A materials showed two more peaks at lower temperatures, i.e., 238–247 °C and 320–321 °C, which are attributed to degradation of the additional features of PUA-A vs. PUA-B, namely the urea bonds towards ethylenediamine.

## 3. Conclusions

Spherical polyurea (PUA) aerogel beads were synthesized at room temperature, without the use of surfactants, from an aliphatic triisocyanate (Desmodur N3300) and ethylenediamine. A propylene carbonate solution of the isocyanate was added dropwise to a mixture of ethylenediamine and heavy mineral oil, and beads were formed at the shape of the drops. The beads had a mean diameter of 2.7 mm and their size distribution was narrow (full width at half maximum: 0.4 mm). To our knowledge, this work comprises the first-time report of uniform, millimeter-size PUA particles via a method that can be easily applied for large-scale production. The material properties of these beads were similar to those of chemically related but morphologically similar monoliths reported in the literature from the reaction of the same triisocyanate with water in acetone, suggesting that nanostructure rather than minor differences in chemical composition is the property-determining factor. SEM showed that the surface of the beads had a different morphology from their interior, which may be explained by the diffusion profile of ethylenediamine in the propylene carbonate droplets.

## 4. Materials and Methods

Propylene carbonate and acetone were purchased from Acros Organics (Geel, Belgium). Ethylenediamine and triethylamine were purchased from Thermo Fisher Scientific (Waltham, MA, USA). Isocyanate Desmodur N3300 was kindly provided by Covestro Deutschland GA (Leverkusen, Germany). Nujol oral (heavy mineral oil; viscosity: 110–230 mPa⋅s) was purchased from Bayer Hellas (Athens, Greece).

^1^H MAS and ^13^C CPMAS NMR spectra were obtained with a 600 MHz Varian spectrometer operating at 150.80 MHz for ^13^C. For ^1^H-^13^C ramped CPMAS spectra, the spinning rate used was 20 kHz and the temperature for running the experiment was 25 °C. FT-IR spectra were obtained on a Shimadzu FT/IR IRAffinity-1 spectrometer (Kyoto, Japan) with samples prepared as KBr pellets. The thermal stability of the materials was studied by TGA, employing a Mettler-Toledo TGA/DSC1 instrument (Schwerzenbach, Switzerland). Samples were placed in alumina crucibles. An empty alumina crucible was used as a reference. Samples were heated from ambient temperatures to 800 °C in a 50 mL/min flow of N_2_ at a heating rate of 10 °C/min. N_2_-sorption measurements were made on a Micromeritics Tristar II 3020 surface area and porosity analyzer (Micromeritics, Norcross, GA, USA). Skeletal densities were determined by He pycnometry, using a Micromeritics AccuPyc II 1340 pycnometer (Micromeritics, Norcross, GA, USA). Bulk density (*ρ_b_*) was calculated from the weight and the mean diameter of the beads, which was calculated using the IBM SPSS Statistics 24. SEM was carried out on free-surface, gold-coated dried aerogel filings, adhered on conductive double-sided adhesive carbon tape, using a Jeol JSM 5600 SEM instrument (Tokyo, Japan).

Supercritical fluid (SCF) drying was carried out in an autoclave (E3100, Quorum Technologies, East Sussex, UK). Wet-gels were placed in the autoclave at 14 °C and were covered with acetone. Liquid CO_2_ was allowed in the autoclave; acetone was drained out as it was being displaced by liquid CO_2_ (5×; 1 per 30 min). Afterwards, the temperature of the autoclave was raised to 45 °C and was maintained for 1 h. Finally, the pressure was gradually released, allowing SCF CO_2_ to escape as a gas, leaving dry-gels (aerogels).

### 4.1. Synthesis of Polyurea (PUA) Aerogel Beads

Heavy mineral oil (25 mL, density = 0.84 g cm^–3^) and ethylenediamine (2.5 mL, 2.25 g, 37.4 mmol) were added into a volumetric cylinder and were mixed vigorously. Desmodur N3300 (9.4 mL, 11 g, 21.8 mmol) was dissolved in propylene carbonate (94 mL). The density of that solution was about 1.3 g cm^–3^, and it was added dropwise through a disposable borosilicate glass Pasteur pipette 1 mm in diameter into the cylinder containing ethylenediamine, at a rate of 0.3 mL min^–1^. The wet-gel spherical beads were formed as soon as the droplets penetrated the surface of the mineral oil. Those wet-gel beads settled at the bottom of the cylinder and were left for aging for 15 min after the end of the addition. Subsequently, the mineral oil solution was decanted and wet-gel beads were solvent-exchanged with acetone (8× their volume; 5 washes, 8 h each), and were dried in an autoclave with supercritical CO_2_.

### 4.2. Synthesis of Polyurea (PUA) Monoliths

Monoliths were synthesized according to the literature procedure [17], using:

(a) for PUA-A monoliths: Desmodur N3300 (9.4 mL, 11 g, 21.8 mmol), ethylenediamine (2.0 mL, 1.8 g, 30.2 mmol) and propylene carbonate (94 mL).
(b) for PUA-B monoliths: Desmodur N3300 (9.4 mL, 11 g, 21.8 mmol), water (1.2 mL, 1.2 g, 66.7 mmol), Et_3_N (0.4 mL, 0.3 g, 2.9 mmol) and propylene carbonate (94 mL).

## Figures and Tables

**Table 1:** Properties of spherical PUA aerogel beads in comparison to PUA monoliths prepared from Desmodur N3300 according to Equation (2).

PUA Sample	Bulk Density *ρ_b_* (g cm^–3^)	Skeletal Density *ρ_s_* (g cm^–3^)	Porosity ^a^ *II* (% *v*/*v*)	BET Surf. Area ^b^ *σ* (m^2^ g^–1^)	*V*_Total_^c^ (*V*_1.7–300nm_) ^d^ (cm^3^ g^−1^)	av. pore diam. ^e^ (4*V*_Total_/*σ*) (nm)	Particle Size ^f^ *r* (nm)
PUA-A beads	0.166 ± 0.001	1.246 ± 0.005	87	197	5.2 (1.2)	24 (105)	12
PUA-B monolith-propylene carbonate	0.110 ± 0.001	1.236 ± 0.006	91	73	8.3 (0.2)	13 (455)	33
PUA-B monolith-acetone ^g^	0.126 ± 0.001	1.265 ± 0.006	90	169	7.1 (1.1)	42 (169)	14

^a^ Calculated according to the formula: (*ρ*_s_ − *ρ*_b_)/*ρ*_s_, where *ρ*_s_: skeletal density and *ρ*_b_: bulk density. ^b^ Brunauer–Emmett–Teller (BET) surface area. ^c^ Volume calculated according to formula: 1/*ρ*_b_ – 1/*ρ*_s_. ^d^ Cumulative volume of pores between 1.7 and 300 nm from N_2_-sorption data and the Barrett–Joyner–Halenda (BJH) desorption method. ^e^ Calculated by the 4*V*/*σ* method; *V* was set equal to the maximum volume of N_2_ adsorbed along the isotherm as *P*/*P*_o_ → 1.0. ^f^ Calculated by the formula: *r* = 3/(*ρ*_s_ × *σ*). ^g^ Values obtained from reference [26].

**Table 2:** Thermal decomposition of PUA spherical aerogel beads and monoliths.

PUA Sample	Start (°C)	End (°C)	1st Peak (°C)	2nd Peak (°C)	3rd Peak (°C)	4th Peak (°C)
PUA-A beads	179.7	510.5	246.7	321.3	381.0	463.0
PUA-A monolith	179.9	510.5	238.4	320.4	382.3	465.8
PUA-B monolith	217.1	508.6	-	-	381.3	464.1

## Supplementary Materials

The following are available online at http://www.mdpi.com/2310-2861/4/3/66/s1, Figure S1: ^13^C CPMAS NMR spectra of PUA spherical aerogel beads prepared according to Equation (1) (PUA-A; bottom spectrum) and monoliths prepared according to Equation (2) (PUA-B; top spectrum).

## References

[1] Pierre A.C., Pajonk G.M. (2002). Chemistry of aerogels and their applications. Chem. Rev..

[2] Aegerter M.A., Leventis N., Koebel M.M. (2011). Aerogels Handbook.

[3] Leventis N., Sadekar A., Chandrasekaran N., Sotiriou-Leventis C. (2010). Click synthesis of monolithic silicon carbide aerogels from polyacrylonitrile-coated 3D silica networks. Chem. Mater..

[4] Vareda J.P., Lamy-Mendes A., Durães L. (2018). A reconsideration on the definition of the term aerogel based on current drying trends. Microporous Mesoporous Mater..

[5] Ziegler C., Wolf A., Liu W., Herrmann A.-K., Gaponik N., Eychmüller A. (2017). Modern inorganic aerogels. Angew. Chem. Int. Ed..

[6] Mohanan J.L., Arachchige I.U., Brock S.L. (2005). Porous semiconductor chalcogenide aerogels. Science.

[7] Rewatkar P.M., Taghvaee T., Saeed A.M., Donthula S., Mandal C., Chandrasekaran N., Leventis T., Shruthi T.K., Sotiriou-Leventis C., Leventis N. (2018). Sturdy, monolithic SiC and Si_3_N_4_ aerogels from compressed polymer-cross-linked silica xerogel powders. Chem. Mater..

[8] Leventis N., Vassilaras P., Fabrizio E.F., Dass A. (2007). Polymer nanoencapsulated rare earth aerogels: Chemically complex but stoichiometrically similar core-shell superstructures with skeletal properties of pure compounds. J. Mater. Chem..

[9] Schäfer H., Milow B., Ratke L. (2013). Synthesis of inorganic aerogels via rapid gelation using chloride precursors. RSC Adv..

[10] Smirnova I., Gurikov P. (2017). Aerogels in chemical engineering: Strategies toward tailor-made aerogels. Annu. Rev. Chem. Biomol. Eng..

[11] Subrahmanyam R., Gurikov P., Dieringer P., Sun M., Smirnova I. (2015). On the road to biopolymer aerogels—Dealing with the solvent. Gels.

[12] Zhao S., Malfait W.J., Guerrero-Alburquerque N., Koebel M.M., Nyström G. (2018). Biopolymer aerogels and foams: Chemistry, properties, and applications. Angew. Chem. Int. Ed..

[13] De France K.J., Hoare T., Cranston E.D. (2017). Review of hydrogels and aerogels containing nanocellulose. Chem. Mater..

[14] Donthula S., Mandal C., Leventis T., Schisler J., Saeed A.M., Sotiriou-Leventis C., Leventis N. (2017). Shape memory superelastic poly (isocyanurate-urethane) aerogels (PIR-PUR) for deployable panels and biomimetic applications. Chem. Mater..

[15] Meador M.A.B., Agnello M., McCorkle L., Vivod S.L., Wilmoth N. (2016). Moisture-resistant polyimide aerogels containing propylene oxide links in the backbone. ACS Appl. Mater. Interfaces.

[16] Kanellou A., Anyfantis G.C., Chriti D., Raptopoulos G., Pitsikalis M., Paraskevopoulou P. (2018). Poly (urethane-norbornene) aerogels via ring opening metathesis polymerization of dendritic urethane-norbornene monomers: Structure-property relationships as a function of an aliphatic versus an aromatic core and the number of peripheral norbornene moieties. Molecules.

[17] Leventis N., Sotiriou-Leventis C., Chandrasekaran N., Mulik S., Larimore Z.J., Lu H., Churu G., Mang J.T. (2010). Multifunctional polyurea aerogels from isocyanates and water. A structure−property case study. Chem. Mater..

[18] Leventis N., Chandrasekaran N., Sadekar A.G., Sotiriou-Leventis C., Lu H. (2009). One-pot synthesis of interpenetrating inorganic/organic networks of CuO/resorcinol-formaldehyde aerogels: Nanostructured energetic materials. J. Am. Chem. Soc..

[19] Mohite D.P., Larimore Z.J., Lu H., Mang J.T., Sotiriou-Leventis C., Leventis N. (2012). Monolithic hierarchical fractal assemblies of silica nanoparticles cross-linked with polynorbornene via ROMP: A structure–property correlation from molecular to bulk through Nano. Chem. Mater..

[20] Leventis N. (2007). Three-dimensional core-shell superstructures: Mechanically strong aerogels. Acc. Chem. Res..

[21] Paraskevopoulou P., Gurikov P., Raptopoulos G., Chriti D., Papastergiou M., Kypritidou Z., Skounakis V., Argyraki A. (2019). Strategies toward catalytic biopolymers: Incorporation of tungsten in alginate aerogels. Polyhedron.

[22] Chen H.-B., Schiraldi D.A. (2018). Flammability of polymer/clay aerogel composites: An overview. Polym. Rev..

[23] De Vos R., Biesmans G.L.J.G. (1996). Organic Aerogels. U.S. Patent.

[24] Moon S.-Y., Jeon E., Bae J.-S., Byeon M., Park J.-W. (2014). Polyurea networks via organic sol–gel crosslinking polymerization of tetrafunctional amines and diisocyanates and their selective adsorption and filtration of carbon dioxide. Polym. Chem..

[25] Yang Y., Jiang X., Zhu X., Kong X.Z. (2015). A facile pathway to polyurea nanofiber fabrication and polymer morphology control in copolymerization of oxydianiline and toluene diisocyanate in acetone. RSC Adv.

[26] Leventis N., Chidambareswarapattar C., Bang A., Sotiriou-Leventis C. (2014). Cocoon-in-web-like superhydrophobic aerogels from hydrophilic polyurea and use in environmental remediation. ACS Appl. Mater. Interfaces.

[27] Leventis N., Sotiriou-Leventis C., Chandrasekaran N., Mulik S., Chidambareswarapattar C., Sadekar A., Mohite D., Mahadik S.S., Larimore Z.J., Lu H. (2011). Isocyanate-derived organic aerogels: Polyureas, polyimides, polyamides. MRS Online Proc. Libr. Arch..

[28] Jones F.N., Nichols M.E., Pappas S.P. (2017). Organic Coatings: Science and Technology.

