# Supplementary material for: Millimeter-Size Spherical Polyurea Aerogel Beads with Narrow Size Distribution
Source: Gels. 2018 Aug 6;4(3):66. doi: 10.3390/gels4030066 (PMC6209287; doi:10.3390/gels4030066)
Supplement: Supplementary file 1 [file gels-04-00066-s001.pdf]

gels

MDPI

Article

Millimeter-Size Spherical Polyurea Aerogel Beads with Narrow Size Distribution

Despoina Chriti, Grigorios Raptopoulos, Maria Papastergiou and Patrina Paraskevopoulou*

Laboratory of Inorganic Chemistry, Department of Chemistry, National and Kapodistrian University of Athens, Panepistimiopolis Zografou, Athens 15771, Greece.

* Correspondence: paraskevopoulou@chem.uoa.gr (P.P.); Tel.: +30-210-727-4381; Fax: +30-210-727-4782

Received: 3 July 2018; Accepted: 3 August 2018; Published: 6 August 2018

SUPPORTING INFORMATION

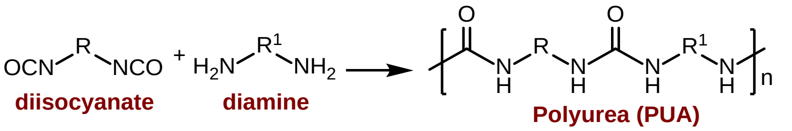

(1)

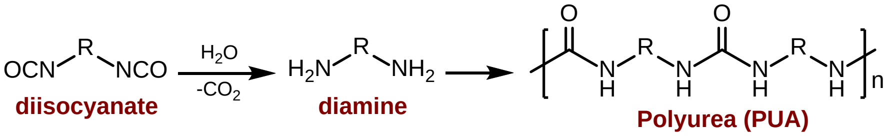

(2)

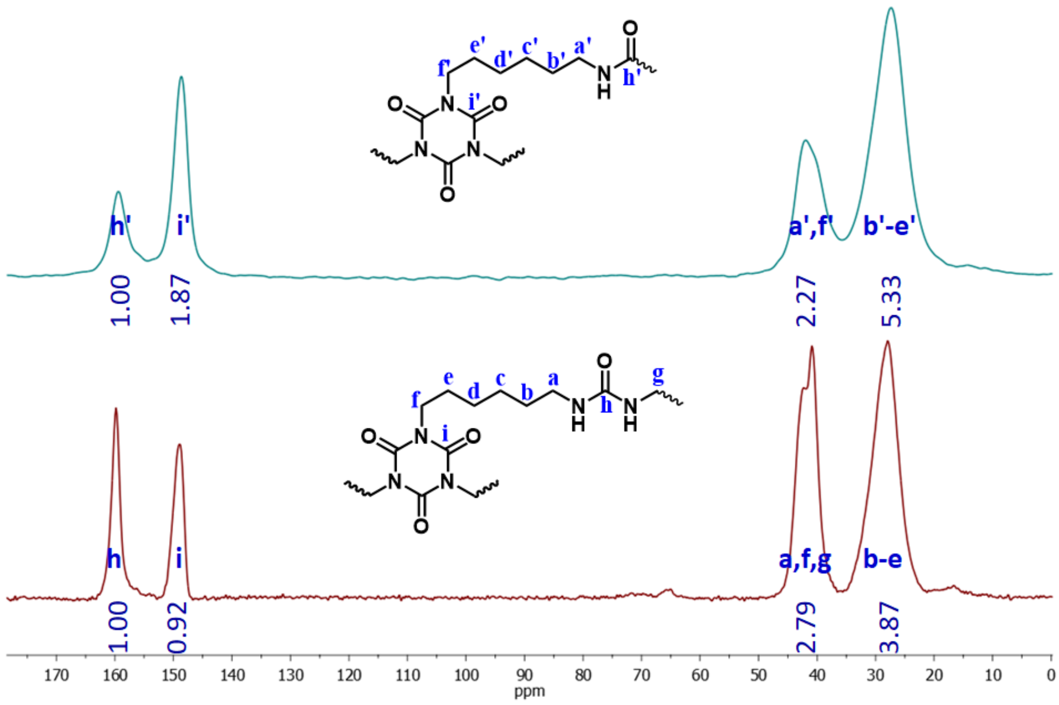

Figure S1. ^{13}C CPMAS NMR spectra of PUA spherical aerogel beads prepared according to Equation 1 (PUA-A; bottom spectrum) and monoliths prepared according to Equation 2 (PUA-B; top spectrum).
